# Fulminant Unilateral Pseudomonas aeruginosa Endogenous Endophthalmitis Secondary to Peripheral Venous Catheter-Related Bacteremia in a Full-Term Neonate: A Case Report

**DOI:** 10.7759/cureus.111460

**Published:** 2026-06-25

**Authors:** Hassan Moutei, Salma Kabbaj, Fouad Chraibi, Meriem Abdellaoui, Idriss Benatiya Andaloussi

**Affiliations:** 1 Department of Ophthalmology, Hassan II University Hospital, Fez, MAR

**Keywords:** bacterial endogenous endophthalmitis, neonatal intensive care unit (nicu), neonatal sepsis, peripheral venous catheter, pseudomonas aeruginosa

## Abstract

Endogenous endophthalmitis is a rare but devastating complication of neonatal sepsis, predominantly reported in premature infants with very low birth weight. We report fulminant unilateral *Pseudomonas aeruginosa* endogenous endophthalmitis in a seven-day-old full-term male neonate (gestational age 39 weeks, birth weight 3,200 g) following delivery complicated by maternal chorioamnionitis and acute fetal distress. The infection originated from a peripheral intravenous catheter-related bloodstream infection, confirmed by triple-concordant cultures of blood, catheter tip, and vitreous aspirate. Ophthalmic examination revealed intense periorbital edema, dense fibrinous hypopyon, diffuse corneal stromal edema, absent red reflex, and a superotemporal subconjunctival collection consistent with a scleral abscess. B-scan ultrasonography demonstrated severe vitritis with maintained retinal attachment. Despite immediate multimodal antimicrobial therapy including intravitreal vancomycin and ceftazidime, fortified topical antibiotics, and escalated systemic therapy, the clinical course was relentlessly progressive, culminating in established phthisis bulbi with complete absence of light perception at six months. This case broadens the recognized epidemiological spectrum of neonatal endogenous endophthalmitis and underscores the imperative for early ophthalmologic consultation in any neonatal intensive care unit-admitted neonate with confirmed bacteremia, irrespective of gestational age.

## Introduction

Endogenous endophthalmitis is a rare but devastating intraocular infection caused by hematogenous dissemination of a systemic pathogen across the blood-ocular barrier (a specialized immune interface that, analogous to the blood-brain barrier, normally shields the eye from circulating pathogens) [1]. In neonates, it represents one of the most severe ophthalmologic emergencies encountered in the neonatal intensive care unit (NICU), consistently associated with irreversible visual loss, including phthisis bulbi (a shrunken, nonfunctional eye), corneal perforation, and the need for ocular evisceration or enucleation [2,3]. Unlike exogenous endophthalmitis, which arises from direct ocular inoculation through trauma or surgery, endogenous endophthalmitis results exclusively from hematogenous seeding in the context of systemic bacteremia or fungemia, characteristically lacking a primary ocular entry wound.

Bacterial pathogens predominate, with gram-negative organisms accounting for the majority of cases. *Pseudomonas aeruginosa* is the most frequently reported and most virulent causative organism, responsible for the majority of invasive neonatal ocular bacterial infections. It causes fulminant intraocular destruction through the secretion of proteolytic enzymes that rapidly dissolve the structural proteins of the cornea, sclera, and retina [4,5]. Other identified pathogens include *Klebsiella pneumoniae*, *Staphylococcus aureus*, Group B *Streptococcus*, and *Candida* species, the latter being particularly prevalent in extremely premature infants with prolonged antibiotic exposure. Reported cases occur almost exclusively in premature infants with very low birth weight (VLBW); endogenous endophthalmitis in full-term neonates is exceptionally rare and poorly characterized [4,5]. Furthermore, definitive microbiological confirmation through concordant multisite cultures is rarely documented [6].

Herein, we report fulminant unilateral *P. aeruginosa* endogenous endophthalmitis in a full-term neonate arising from peripheral venous catheter-related bacteremia confirmed by triple-concordant cultures, with the aim of broadening the recognized epidemiological spectrum of this condition and reinforcing the importance of early ophthalmologic consultation in any NICU-admitted neonate with systemic sepsis regardless of gestational age.

## Case presentation

A seven-day-old male neonate born at full term (gestational age 39 weeks, birth weight 3,200 g, Apgar scores 6 and 8 at one and five minutes) was admitted to the NICU following delivery complicated by suspected maternal chorioamnionitis and acute fetal distress. The postnatal period was complicated by transient respiratory depression requiring brief positive-pressure ventilation. A peripheral intravenous catheter was placed in the left upper limb, and empirical intravenous antibiotic therapy with a third-generation cephalosporin was initiated.

On day 7 of life, the neonate developed fever (38.5°C), progressive left eyelid edema, and ocular redness. The catheter insertion site showed erythema, induration, and purulent discharge, raising suspicion for catheter-related bloodstream infection. The infant was urgently referred to ophthalmology for suspected endogenous endophthalmitis.

A bedside ophthalmic examination under sterile conditions was performed. The right eye was unremarkable, with clear cornea, deep quiet anterior chamber, normal intraocular pressure (IOP), and bright red reflex. Posterior segment examination was normal.

**Figure 1:**
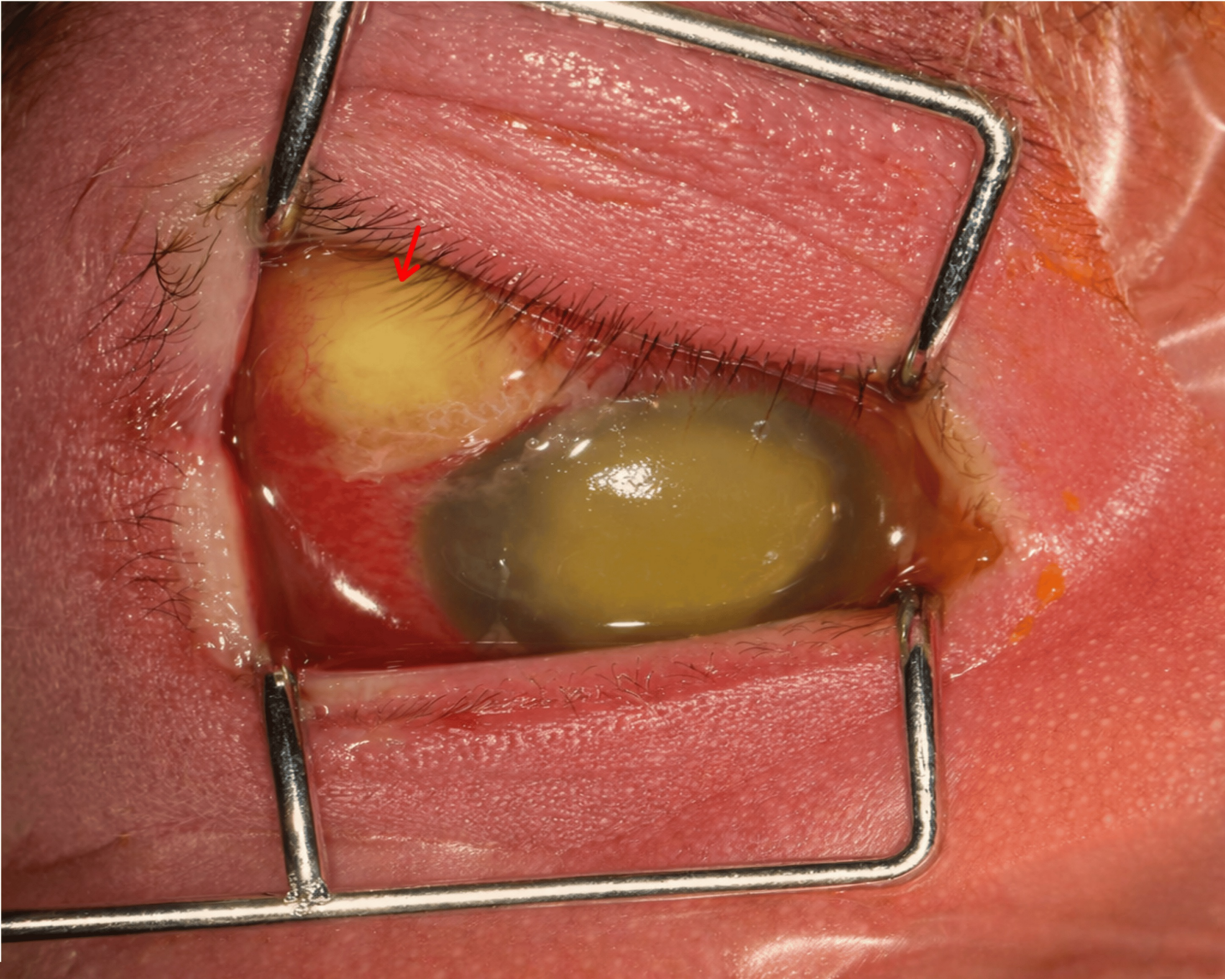
Anterior segment photograph of the left eye demonstrating fulminant endogenous endophthalmitis at presentation Anterior segment photograph of the left eye at presentation showing marked periorbital erythema, eyelid edema, severe conjunctival injection with chemosis, a localized superotemporal subconjunctival collection consistent with scleral abscess (red arrow), diffuse corneal stromal edema with loss of transparency, and dense fibrinous exudate filling the anterior chamber, consistent with fulminant endogenous endophthalmitis.

The left eye demonstrated intense periorbital erythema, pronounced eyelid edema, severe conjunctival injection with chemosis (conjunctival swelling producing a gelatinous appearance of the ocular surface), and adherent purulent secretions. A superotemporal subconjunctival collection consistent with a scleral abscess was identified. The cornea showed diffuse stromal edema with loss of transparency. The anterior chamber was entirely filled with dense fibrinous exudate and hypopyon (a visible layering of pus forming a white fluid level at the base of the anterior chamber), precluding visualization of posterior structures. The red reflex was absent, indicating complete loss of light transmission through the ocular media (Figure 1).

**Figure 2:**
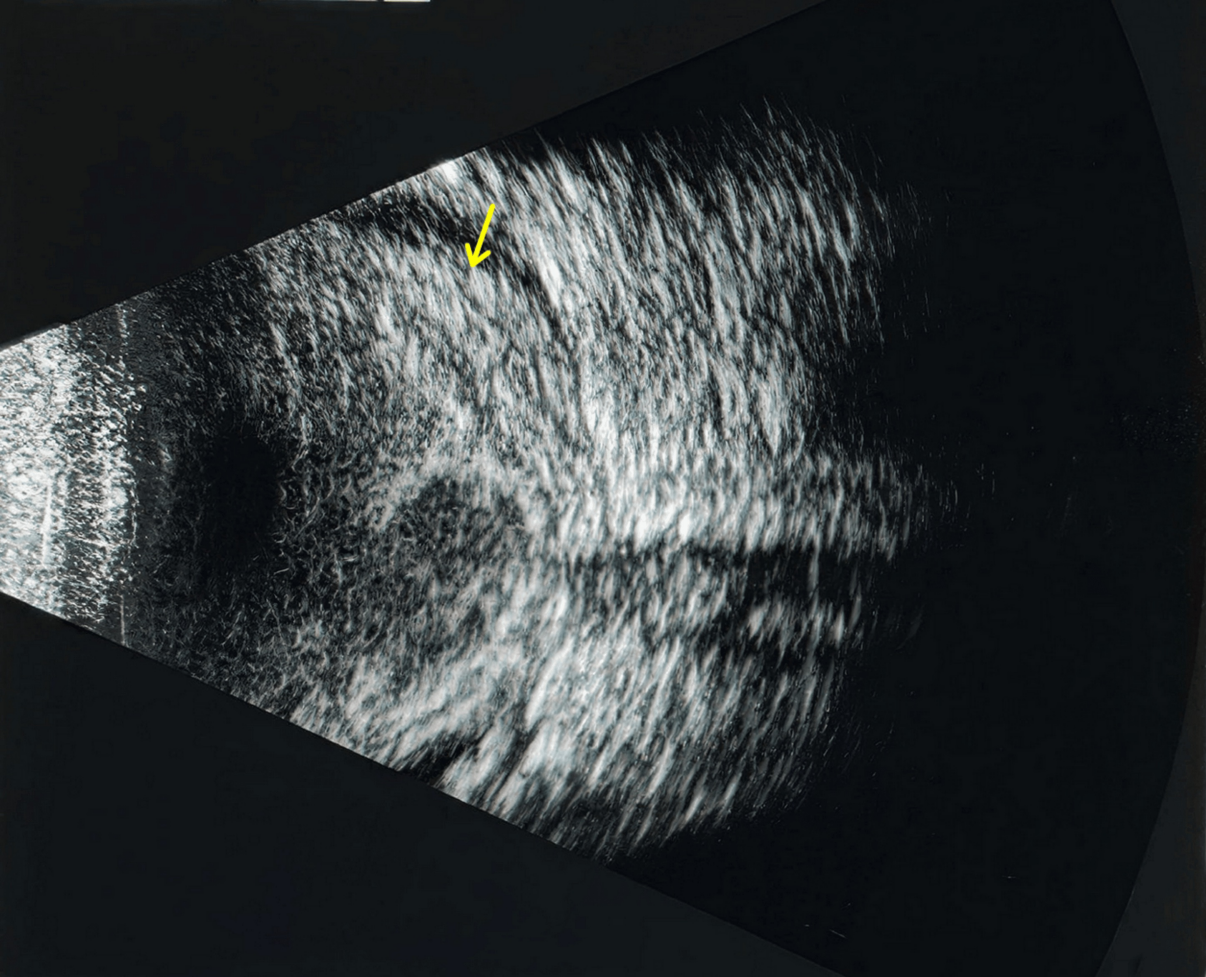
B-scan ultrasonography of the left eye at presentation B-scan ultrasonography of the affected left eye demonstrating markedly hyperechogenic vitreous with dense membranous echoes distributed throughout the vitreous cavity, consistent with severe vitritis (yellow arrow), with the retina remaining attached in all four quadrants.

B-scan ultrasonography of the left eye revealed markedly hyperechogenic vitreous consistent with severe vitritis, with the retina remaining attached in all four quadrants. Axial length measured 16.5 mm versus 17.0 mm in the right eye (Figure 2).

A diagnostic vitreous tap yielded 0.05 mL of xanthochromic fluid. Gram staining showed numerous neutrophils without visible microorganisms. Vitreous culture grew *P. aeruginosa* after 48 hours. Blood cultures and catheter tip culture grew the identical organism, confirming catheter-related bacteremia with hematogenous intraocular dissemination. Urine culture and cerebrospinal fluid (CSF) analysis were negative; CSF parameters were normal. Complete blood count revealed leukocytosis (18,000 cells/μL, 75% neutrophils) and C-reactive protein of 60 mg/L. Liver and renal function were normal.

The final diagnosis was unilateral *P. aeruginosa* endogenous endophthalmitis. Exogenous endophthalmitis, orbital cellulitis, and neonatal conjunctivitis were excluded on clinical, microbiological, and ultrasonographic grounds.

Management consisted of immediate multimodal antimicrobial therapy. Intravitreal vancomycin (1.0 mg/0.1 mL) and ceftazidime (2.25 mg/0.1 mL) were administered following the vitreous tap. Fortified topical ceftazidime 5% and vancomycin 5% were instilled every two hours, with atropine 0.5% three times daily for cycloplegia. Systemic therapy was escalated to intravenous ceftazidime 50 mg/kg every eight hours and ciprofloxacin 10 mg/kg every 12 hours. The infected catheter was removed. Pars plana vitrectomy was discussed but deferred owing to systemic instability precluding prolonged general anesthesia.

**Figure 3:**
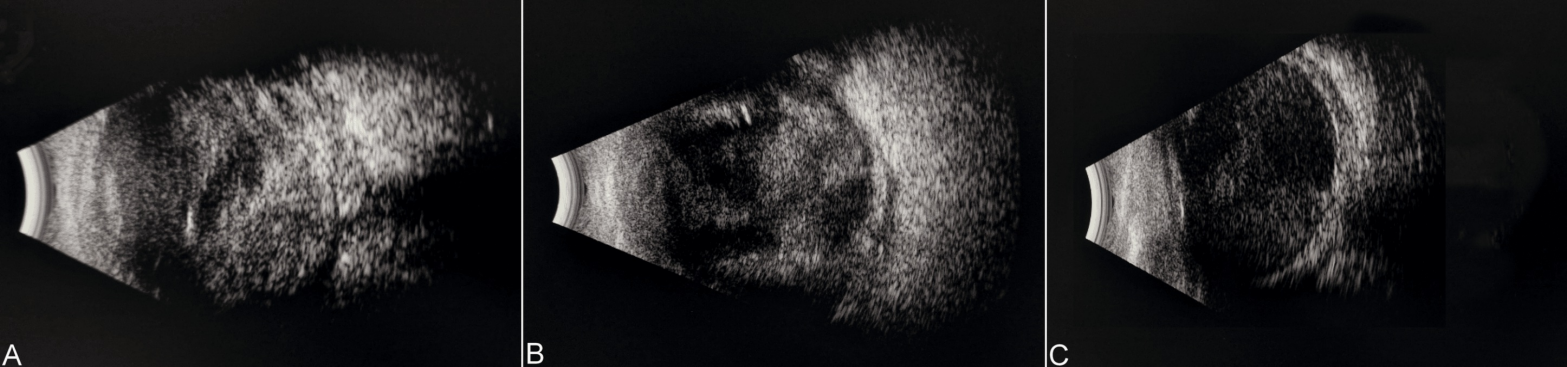
Serial B-scan ultrasonography of the left eye demonstrating progressive intraocular disorganization from acute vitritis to established phthisis bulbi (A) Day 3 after presentation: markedly hyperechogenic vitreous with early dense membranous echo organization, consistent with active vitritis; the retina remains attached in all four quadrants. (B) Day 7 after presentation: progressive organization of vitreous echoes with thickening of membranous formations and persistent diffuse vitritis; retinal attachment is maintained. (C) Six weeks after presentation: marked reduction in globe size with diffuse choroidal thickening, consistent with evolving phthisis bulbi.

Despite an initial modest reduction in periorbital edema and conjunctival hyperemia, corneal edema and anterior chamber fibrin persisted. Serial B-scan ultrasonography on days 3 and 7 showed progressive vitreous organization with dense membranous echoes; the retina remained attached. Over subsequent weeks, the left globe became progressively smaller and hypotonic (Figure 3).

**Figure 4:**
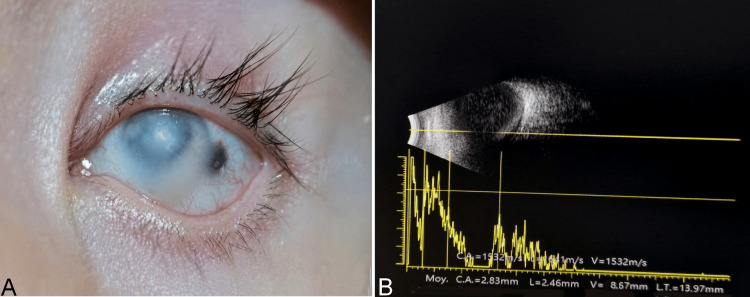
Clinical and biometric documentation of established phthisis bulbi at 6-month follow-up (A) Appearance of the left eye at 6-month follow-up demonstrating a small, shrunken globe with diffuse dense corneal opacification and complete loss of anterior segment visualization, consistent with established phthisis bulbi. (B) A/B-scan biometry at 6-month follow-up confirming axial length reduction to 13.97 mm in the affected left eye, with concurrent intraocular structural collapse, consistent with established phthisis bulbi.

At the six-month follow-up, established phthisis bulbi was confirmed: axial length 14.0 mm, IOP 4 mmHg, dense corneal opacification, total posterior synechiae, and absence of light perception (Figure 4). The right eye remained normal throughout. The family was counseled on the definitive visual prognosis and the need for early prosthetic rehabilitation to preserve orbital growth and facial symmetry.

## Discussion

This case documents fulminant *P. aeruginosa* endogenous endophthalmitis in a full-term neonate, confirmed by triple-concordant cultures of blood, catheter tip, and vitreous. Despite maximal multimodal therapy, the clinical course progressed inexorably to established phthisis bulbi. Two features distinguish this report: the epidemiological atypicality of a full-term host and the exceptional degree of microbiological certainty.

Neonatal endogenous endophthalmitis almost exclusively affects premature VLBW infants, in whom immune immaturity, increased blood-ocular barrier permeability, and a highly vascularized developing retina amplify susceptibility [1]. Epidemiologically, the condition is rare, with the largest published cohort comprising 109 affected neonates [6]. Unlike exogenous endophthalmitis, which results from direct ocular inoculation through trauma or surgery, endogenous endophthalmitis arises exclusively from hematogenous seeding during systemic bacteremia or fungemia, characteristically lacking a primary ocular wound. The present case demonstrates that full-term neonates are not immune when invasive NICU procedures and nosocomial bacteremia converge, as illustrated by the concurrent presence of maternal chorioamnionitis, perinatal immune perturbation, and peripheral venous catheterization. Clinicians must therefore maintain vigilance for this diagnosis irrespective of gestational age.

Among causative organisms, gram-negative bacteria predominate. *P. aeruginosa* is the most frequently reported pathogen and is consistently associated with the most fulminant outcomes [6,7]. Other identified pathogens include *K. pneumoniae*, *S. aureus*, Group B *Streptococcus*, and *Candida* species. Its exceptional virulence is attributable to proteolytic enzymes that rapidly dissolve the structural proteins of the cornea, sclera, and retina, rendering visual prognosis uniformly poor. Although central venous catheters are the most frequently implicated infection source, this case highlights peripheral intravenous catheters as an underemphasized portal of entry [2,7,8]. Vitreous cultures may yield false-negative results due to prior antibiotic exposure or low specimen volume; triple-concordant cultures here established an unambiguous hematogenous pathway and provided early microbiological guidance before culture results were available [6,7].

B-scan ultrasonography was the primary diagnostic tool, confirming severe vitritis with maintained retinal attachment [9]. The subtle axial length asymmetry at presentation (16.5 mm versus 17.0 mm) may represent an early ultrasonographic marker of global compromise. Serial imaging documented progression from active vitritis to vitreous organization and ultimately phthisis, consistent with published descriptions of the condition's natural history.

Multimodal therapy combining intravitreal vancomycin and ceftazidime, fortified topical antibiotics, and systemic ceftazidime with ciprofloxacin adhered to established management principles [6,10]. The doses used reflect adult-based guidelines, as no neonatal-specific intravitreal dosing recommendations currently exist. Given the smaller neonatal globe volume, effective intraocular drug concentration is substantially higher, raising theoretical retinal toxicity concerns with repeat injections. Pars plana vitrectomy, which reduces microbial burden and improves antibiotic penetration, was deferred owing to systemic instability [10,11]. Given the narrow therapeutic window with *Pseudomonas*, expedited vitrectomy as soon as systemic stability permits warrants serious consideration in future comparable cases.

The principal strengths of this report are its robust triple-concordant microbiological documentation and detailed serial ultrasonographic characterization from acute presentation through established phthisis. Limitations include the inability to generalize from a single case and the absence of vitrectomy, precluding assessment of its potential impact. The small vitreous volume (0.05 mL) constrained microbiological testing breadth.

## Conclusions

*P. aeruginosa* endogenous endophthalmitis can affect full-term neonates and carries a uniformly devastating prognosis despite maximal multimodal therapy. Peripheral venous catheters represent a clinically relevant infection source requiring meticulous management. Early ophthalmologic consultation in any bacteremic NICU-admitted neonate, prompt source control, and expedited vitrectomy when systemically feasible should be considered the standard of care. Systematic ophthalmologic screening of neonates with confirmed bloodstream infections merits prospective evaluation as a strategy to reduce diagnostic delay and potentially improve outcomes.
